# Body composition and cardiorespiratory fitness of overweight COVID-19 survivors in different severity degrees: a cohort study

**DOI:** 10.1038/s41598-023-44738-8

**Published:** 2023-10-17

**Authors:** Victor Augusto Santos Perli, Ana Flávia Sordi, Maurício Medeiros Lemos, Jhemilly Scarleth Araujo Fernandes, Virgínia Benedetti Nanuncio Capucho, Bruno Ferrari Silva, Solange de Paula Ramos, Pablo Valdés-Badilla, Jorge Mota, Braulio Henrique Magnani Branco

**Affiliations:** 1 University Cesumar, Maringa, Parana Brazil; 2 Graduate Program in Health Promotion, University Cesumar, Maringa, Paraná Brazil; 3 State University of Londrina, Londrina, Parana Brazil; 4 Department of Physical Activity Sciences, Faculty of Education Sciences, Universidad Católica del Maule, Talca, Chile; 5 Sports Coach Career, School of Education, Universidad Viña del Mar, Viña del Mar, Chile; 6 Laboratory for Integrative and Translational Research in Population Health (ITR), Research Center of Physical Activity, Health, and Leisure, Faculty of Sports, University of Porto, Porto, Portugal; 7 Interdisciplinary Laboratory of Intervention in Health Promotion, Cesumar Institute of Science, Technology and Innovation, Avenida Guedner, 1610, Maringá, Paraná Brazil

**Keywords:** Public health, Quality of life, Weight management, Body mass index, Cardiology, Risk factors

## Abstract

COVID-19 sequelae are varied, and whether they are temporary or permanent is still unknown. Identifying these sequelae may guide therapeutic strategies to improve these individuals' recovery. This prospective cohort aimed to assess body composition, cardiopulmonary fitness, and long-term symptoms of overweight individuals affected by COVID-19. Participants (*n* = 90) were divided into three groups according to the severity of acute COVID-19: mild (no hospitalization), moderate (hospitalization, without oxygen support), and severe/critical cases (hospitalized in Intensive Care Unit). We assessed body composition with a tetrapolar multifrequency bioimpedance, hemodynamic variables (heart rate, blood pressure, and peripheral oxygen saturation-SpO_2_) at rest, and the Bruce test with direct gas exchange. Two assessments with a one-year interval were performed. The most prevalent long-term symptoms were memory deficit (66.7%), lack of concentration (51.7%), fatigue (65.6%), and dyspnea (40%). Bruce test presented a time effect with an increase in the distance walked after 1 year just for severe/critical group (*p* < 0.05). SpO_2_ was significantly lower in the severe/critical group up to 5 min after the Bruce test when compared to the mild group, and diastolic blood pressure at the end of the Bruce test was significantly higher in the severe/critical group when compared to mild group (*p* < 0.05; for all comparisons). A time effect was observed for body composition, with increased lean mass, skeletal muscle mass, fat-free mass, and lean mass just for the severe/critical group after 1 year (*p* < 0.05). Cardiopulmonary fitness parameters did not differ among the groups, except for respiratory quotient with higher values for the severe/critical group when compared to itself after 1 year. All COVID-19 patients might present long-term sequelae, regardless of the acute disease severity. Reassessing and identifying the most prevalent long-term sequelae are essential to perform more precise health promotion interventions.

## Introduction

Studies have emphasized that COVID-19 is a respiratory and multisystem syndrome^1,2^. The persistent symptoms of COVID-19 at the end of the acute infectious response, like other viral disorders, are characterized as chronic symptomatic disorders that mainly affect the respiratory, cardiovascular, neurocognitive, digestive, and muscular systems^3^. These symptoms, characterized in the literature as "long COVID", are present in many of the population, ranging from 10 to 35% of those who contracted the disease^4^. The long COVID can present in different forms according to each individual's environmental, physiological, and lifestyle characteristics, such as age, sex, ethnicity, functional activity, social exposure, hospitalization factors, and chronic diseases^5^.

The persistent symptoms of long COVID have been classified into two categories^1^. The first classification encompasses long-term tissue damage mainly affecting the heart, lung, and neurological tissues^6^. The second classification focuses on chronic inflammation leading to viral persistence, lymphopenia, intestinal dysbiosis, and autoimmunity^1,5^. In addition, the effects of long COVID can be social, physical, and mental, generally reducing the quality of life and health of those infected by SARS-CoV-2. Fatigue, difficulty breathing, reduced activities of daily living, and memory problems are frequent sequelae reported in patients after acute infection^4,7^.

Studies have shown that the sequelae of long COVID are a public health problem that implies the indispensability of developing strategies for controlling and caring for these patients^8–10^. In addition, patients with severe acute COVID-19 who require admission to the intensive care unit (ICU) generally showed a higher fat mass, lower cardiorespiratory fitness, myopathies, and neuropathies^6,9^. Given the multifactorial effects, monitoring the clinical evolution of patients has become essential in unraveling the evolution of long COVID^3,10^. This follow-up is indispensable given that physical fitness is vital to performing daily living activities and obtaining good working conditions since the deleterious effects of long COVID are unknown in different organ systems until the present moment^6–10^.

Preventing and treating the physical domains impaired by the syndrome is essential^9^. Among them, monitoring cardiopulmonary and neuromuscular systems could promote aspects that help health professionals during rehabilitation^9,11^. At the best of our knowledge, few studies have assessed the recovery of COVID-19 sequelae after a first diagnostic assessment. Given this, the present study aimed (1) to evaluate body composition, cardiopulmonary fitness, and long symptoms of individuals affected by COVID-19; (2) to compare the features according to the severity of acute disease; and (3) to assess cardiopulmonary and hemodynamic recovery post stress-test after 1 year. We hypothesize that individuals who presented with more severe acute disease have a slower recovery of cardiopulmonary fitness and long-term symptoms.

## Materials and methods

### Study design

According to the recommendations of the Strengthening the Reporting of Observational Studies in Epidemiology (STROBE) guidelines, this was an observational, longitudinal, and prospective study^12^. We assessed body composition, cardiorespiratory fitness, and persistent symptoms of COVID-19 at two time points with a one-year interval. Participants were allocated into three groups according to disease severity: mild group (no hospitalization), moderate group (hospitalization inward care only), and severely/critically ill group (hospitalized in ICU). The evaluations were conducted at the Exercise Physiology Laboratory of the Higher Education Institution between August and December 2021 and between August and December 2022. In this period, no interventions were performed. Both assessments followed the same procedures. The participants fulfilled a detailed anamnesis with personal information, previous medical history, continuous use of medication, persistent symptoms after COVID-19, and if applied, time of hospitalization and respiratory support modalities required during hospital care.

### Participants

**Figure 1:**
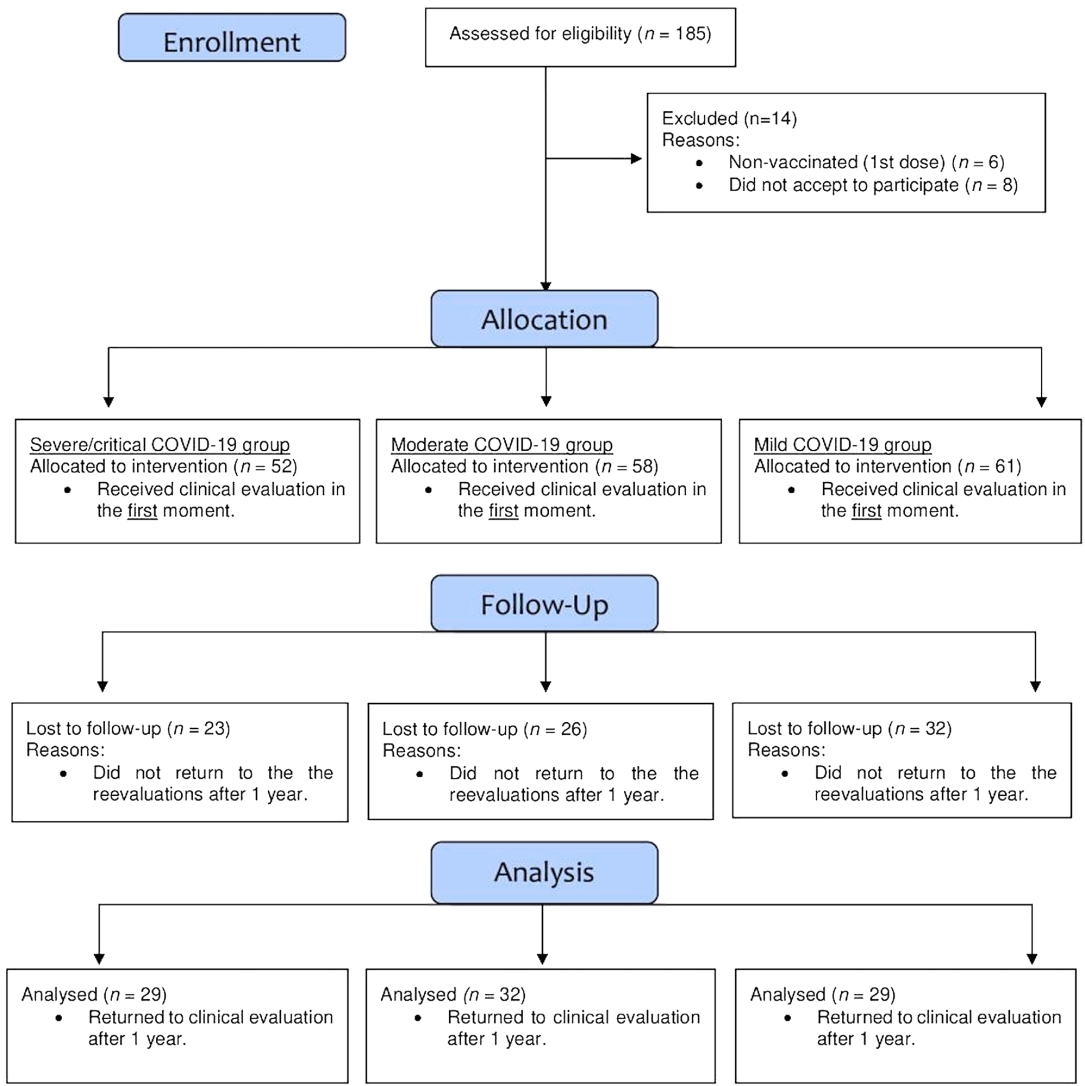
Flowchart of the present study.

One hundred and seventy-one patients (52 with severe/critical cases of COVID-19, 58 moderate cases, and 61 mild cases) were evaluated in 2021 (baseline). After 1 year, 90 patients were re-evaluated: 29 severe/critical cases, 32 moderate cases, and 29 mild cases. Eighty-one patients did not return for reevaluations. Figure 1 shows the flowchart of the present study. COVID-19 symptoms classified patients according to the Independent Oversight Advisory Interim Report on Wealth Health Organization (WHO's) response to COVID-19^13^. Inclusion criteria were: (i) all patients with a positive diagnosis of COVID-19 by reverse transcription-polymerase chain reaction testing (RT‒PCR) between January and July 2021; (ii) aged between 18 to 65 years; (iii) who were overweight or obese classified by body mass index (≥ 25.00 kg/m^2^)^14^; (iv) who received at least one dose of the vaccine against COVID-19; and (v) who received medical clearance to perform the stress test. Exclusion criteria were (i) individuals with disabling neurological disease and reduced mobility or (ii) another physical condition that could affect the performance of the Bruce test.

The sample size was calculated in the G*Power software version 3.1 using the repeated measures analysis of variance (ANOVA) test, with an estimate of the effect based on the values of peak oxygen consumption (VO_2_ peak) found in a previous study with long COVID-19 patients^9^, considering a significance level of *α* = 0.05 and a correlation between repeated measures of 0.5; a sample size of 90 individuals was estimated for *β* = 80%. All participants were informed about the study's aims and possible risks and benefits. They were guaranteed confidentiality and invited to sign the free and informed consent form. The Local Research Ethics Committee approved the study under number 4.546.726/2021, according to resolution 466/2012. The study was conducted according to the provisions of the Declaration of Helsinki.

### Baseline and follow-up assessments

All assessments were performed over two days, with a 24-h interval. A medical clearance, anthropometry, and body composition were performed on the first day. Twenty-four hours later, if the patients were eligible, the Bruce test with direct gas analyzer was performed with monitoring of systolic and diastolic blood pressure (SBP and DBP, mmHg), heart rate (HR, bpm) and peripheral oxygen saturation (SpO_2_, %) up to 15 min after the test according to a previous study^9^. All assessments (baseline and after 1 year) were conducted by the same technical team (with four researchers), which presented an intraclass coefficient correlation of 0.96 to 0.99.

All anamnesis data were self-reported by the patients. The following information was collected: (i) sociodemographic data; (ii) previous morbid history and continuous use of medication; (iii) characteristics of SARS-CoV-2 infection (symptoms, hospital length of stay, and respiratory support modalities during hospitalization); (iv) persistent symptoms after COVID-19 infection; (v) lifestyle habits; and (vi) self-reported physical activity routine.

### Anthropometry and body composition

Height was measured using a Sanny stadiometer (model ES 2030, São Bernardo do Campo, São Paulo, Brazil), measuring 2.20 m with an accuracy of 0.1 cm. Body mass was measured using a Welmy mechanical scale (Model 104A, Santa Bárbara d'Oeste, São Paulo, Brazil), with a capacity of 300 kg and accuracy of 100 g. Body composition was analyzed by the bioelectrical impedance analysis used with an INBODY 570 device (Biospace Co. Ltd., Seoul, Korea), which has eight tactile points, 250 kg capacity, and 100 g precision.

The variables analyzed were body mass (kg), lean mass (LM, in kg), skeletal muscle mass (SMM, in kg), fat mass (FM, in kg), fat-free mass (FFM, in kg), and body fat (BF). The following equation was used to calculate the body mass index (BMI): body mass (kg)/height (m)^2^. The participants were previously instructed to fast for 4 h, not to ingest (caffeine and water), to stop drinking alcohol for at least two days before, not to practice physical exercise for at least 24 h earlier, to urinate 30 min before, and not to wear metal accessories on the body during assessments^15^.

### Bruce’s protocol with vital signs monitoring

The stress test was performed using an INBRAMED treadmill (model ATL 24, Porto Alegre, Rio Grande do Sul) according to the Bruce protocol^16^. First, the vital signs at rest were measured: HR (H10 model, Polar, Kempele, Finland), SpO_2_ (ALFAMED, sense 10 model, Lagoa Santa, Minas Gerais, Brazil) positioned on the index finger, and blood pressure with a stethoscope and sphygmomanometer (PREMIUM brand, standard model, São Paulo, Brazil). All vital signs were measured according to the recommendations of the VIII Brazilian Guideline on Arterial Hypertension^17^.

The tests were performed with complete monitoring of a multidisciplinary team (physicians, nurses, and physical education professionals with specialization in exercise physiology), continuous monitoring of HR, SpO_2_, and measurement of the patient's rating of perceived exertion (RPE) using the Borg scale (6–20 points)^18^. All participants were previously instructed to answer the RPE on the Borg scale. The distance walked in meters (m) during the test was also recorded since the test time could not be used because, in 2021, at least 20% of the patients performed the Adapted Bruce test. The exercise test was terminated in the following cases: (i) voluntary withdrawal of the participant; (ii) RPE ≥ 19; (iii) respiratory quotient (RQ) > 1.15; (iv) signs of exercise intolerance such as lower limb fatigue; and (v) physical inability to maintain intensity during the test.

Gas exchange was analyzed using a VO2000 device (MEDGRAPHICS Corp., Saint Paul, United States of America), with measurement of VO_2_ (L/min) and VCO_2_ (L/min), minute-ventilation (VE, L/min), oxygen pulse (VO_2_/HR), RQ and HR, via a Polar H10 heart rate monitor (POLAR, Kempele, Finland). The equipment was calibrated at each ergo spirometric analysis according to the standardization recommendation^9^. The peak relative values for VO_2_ and VCO_2_ were used for the statistical analyses. Peak values were determined by the highest values obtained during the stress test. The laboratory room temperature was standardized at 24 °C.

### Monitoring of vital signs

At the end of the Bruce test, the patients were placed in a quiet environment. HR and SpO_2_ were monitored minute by minute for 15 min. Systolic blood pressure (SBP) and diastolic blood pressure (DBP) were measured immediately after the exercise and every 5 min for the next 15 min by the same evaluators at both measurement times, i.e., 2021 and 2022^9^.

### Statistical analysis

The distributions of the continuous variables were analyzed using skewness, kurtosis tests, and visual inspection of the histograms. Data with a normal distribution are described as the mean ± standard deviation. Data with a non-normal distribution is described as the median and percentiles (25–75%). Categorical data were expressed as absolute (*n*) and relative frequency (%). A significance of 5% was established for all statistical tests (*p* < 0.05). To analyze possible differences between the characteristics of the three groups, we used the one-way ANOVA for the continuous variables and the chi-square test (χ^2^) for the categorical variables. A repeated two-way ANOVA was used for the stress test performance and body composition variables with time (baseline *vs* 1 year), group (mild, moderate, and severe/critical group), and time-group interaction effects. An ANOVA was also used to analyze the delta variability in post-Bruce test vital signs (from 1 to 15 min after the end of the test). Post-hoc Bonferroni correction was used for all ANOVA analyses when a significant difference was found. A paired t-test (before *vs* after 1 year) was applied when a time difference was identified to verify potential statistical significance in intra-group conditions, and the confidence interval (CI) was also calculated^19^. The partial effect size eta square (ƞ^2^_p_) was classified according to Richardson^20^: 0.0099 (*small effect*), 0.0588 (*moderate effect*), and 0.1379 (*large effect*). Cohen's *d* was calculated and classified following the classification: 0.2 (*small effect*), 0.5 (*moderate effect*), and 0.8 (*large effect*)^21^. Statistical analyses were performed using JAMOVI software, version 1.6.23.

### Ethical approval

This study was conducted according to the guidelines of the Declaration of Helsinki and approved by the Scientific Ethics Committee of University Cesumar (approval number: No. 4.546.726/2021). Informed consent was obtained from all subjects involved in the study.

## Results

### Sample characteristics

Table 1 presents the participants' characteristics according to each group. No significant differences existed between the groups for any aspects (*p* > 0.05), except for the length of hospitalization (*p* < 0.05), with more days of hospitalization for the severe group when compared to the other groups.

**Table 1:** Characteristics of the study participants.

Variables	Groups		
	Mild (*n* = 29)	Moderate (*n* = 32)	Severe/critical (*n* = 29)
Age (years old)	48.4 ± 11.2	50.3 ± 13.9	51.2 ± 10.7
Sex*			
Male, n (%)	15 (51.7)	16 (50.0)	16 (55.2)
Female, n (%)	14 (48.3)	16 (50.0)	13 (44.8)
BMI (kg/m^2^)	28.6 ± 5.1	30.6 ± 5.8	31.7 ± 4.9
Length of hospitalization			
Total (days)	–	7.0 (6.0–10.0)	23.0 (11.0–35.0)*
Ward (days)	–	7.0 (6.0–10.0)	9.5 (4.8–13.3)
ICU (days)	–	–	15.0 (4.8–23.0)
Medical history			
SAS, n (%)	5 (17.2)	13 (40.6)	12 (41.4)
Type 2 diabetes mellitus, n (%)	4 (13.8)	3 (9.4)	3 (10.3)
Dyslipidemia, n (%)	5 (17.2)	9 (28.1)	9 (31.0)
CAD/Revascularization, n (%)	2 (6.9)	0 (0.0)	4 (13.8)
COPD, n (%)	0 (0.0)	0 (0.0)	0 (0.0)
Asthma, n (%)	1 (3.4)	1 (3.1)	0 (0.0)
Smoking			
No, n (%)	20 (71.4)	26 (83.9)	22 (78.6)
Former or current, n (%)	8 (28.6)	5 (16.1)	6 (21.4)
Regular physical activity^§^			
Currently, n (%)	12 (41.4)	9 (28.1)	13 (44.8)
Before COVID-19, n (%)	11 (37.9)	10 (31.3)	15 (51.7)
Baseline vital signs			
HR (bpm)	74 ± 12	76 ± 11	74 ± 9
SBP (mmHg)	118.3 ± 12.3	124.4 ± 15.4	124.6 ± 16.2
DBP (mmHg)	77.9 ± 10.1	83.6 ± 11.5	80.6 ± 9.0
SpO_2_ (%)	97.0 ± 1.4	97.3 ± 1.5	97.4 ± 1.2

Continuous data are presented as mean ± standard deviation or median and percentiles 25–75; categorical data are presented as absolute (n) and relative (%) frequency; AMI = Acute Myocardial Infarction; BMI = Body Mass Index; CAD = Coronary Artery Disease; COPD = Chronic Obstructive Pulmonary Disease; DBP = Diastolic Blood Pressure; HR = Heart Rate; SAS = Systemic arterial hypertension; SBP = Systolic Blood Pressure; SpO_2_ = Peripheral Oxygen Saturation; * = sex was classified according to the biological, physical and physiological features, designated at birth; ^§^ = physical activity considered regular when ≥ 150 min/week; * = a significant difference with more days of hospitalization when compared to the other groups.

### Long-term symptoms

Table 2 presents self-reported persistent long COVID symptoms in the second time point. The most prevalent symptoms were memory deficit (66.7%), fatigue (65.6%), difficulty concentrating (51.7%), and dyspnea (40.0%). No significant differences were found in the groups (*p* > 0.05).

**Table 2:** Long COVID symptoms self-reported by the participants in the 2022 assessments.

Variables	Groups			
	Mild (*n* = 29)	Moderate (*n* = 32)	Severe/critical (*n* = 29)	Total (*n* = 90)
Fatigue, n (%)	19 (65.5)	22 (68.8)	18 (62.1)	59 (65.6)
Small effort, n (%)	7 (24.1)	3 (9.4)	3 (10.3)	13 (14.4)
Moderate effort, n (%)	6 (20.7)	10 (31.3)	6 (20.7)	22 (24.4)
Greater effort, n (%)	6 (20.7)	9 (28.1)	9 (31.0)	24 (26.7)
Dyspnea, n (%)	12 (41.4)	12 (37.5)	12 (41.4)	36 (40.0)
Small effort, n (%)	2 (6.9)	1 (3.1)	1 (3.4)	4 (4.4)
Moderate effort, n (%)	2 (6.9)	6 (18.8)	6 (20.7)	14 (15.6)
Great effort, n (%)	8 (27.6)	5 (15.6)	5 (17.2)	18 (20.0)
Muscle pain, n (%)	8 (27.6)	10 (31.3)	8 (27.6)	26 (28.9)
Joint pain, n (%)	10 (34.5)	6 (18.8)	10 (34.5)	26 (28.9)
Headache, n (%)	8 (27.6)	9 (28.1)	5 (17.2)	22 (24.4)
Dizziness, n (%)	9 (31.0)	12 (37.5)	9 (31.0)	30 (33.3)
Tinnitus, n (%)	6 (20.7)	8 (25.0)	10 (34.5)	24 (26.7)
Sensation of hearing loss, n (%)	7 (24.1)	7 (21.9)	5 (17.2)	19 (21.1)
Otalgia, n (%)	5 (17.2)	5 (15.6)	3 (10.3)	13 (14.4)
Ageusia, n (%)	5 (17.2)	6 (18.8)	5 (17.2)	16 (17.8)
Anosmia, n (%)	5 (17.2)	4 (12.5)	5 (17.2)	14 (15.6)
Memory deficit, n (%)	20 (69.0)	22 (68.8)	18 (62.1)	60 (66.7)
Difficulty concentrating, n (%)	17 (58.6)	16 (50.0)	13 (46.4)	46 (51.7)
Capillary loss, n (%)	7 (24.1)	15 (46.9)	9 (31.0)	31 (34.4)

Data are presented as absolute (*n*) and relative (%) frequency.

### Body composition

Table 3 presents the participants' body mass, BMI, and body composition.

**Table 3:** Anthropometric and body composition parameters of the study participants.

Variables	Mild (*n* = 29)		Moderate (*n* = 32)		Severe/critical (*n* = 29)	
	Before	After	Before	After	Before	After
BM (kg)	79.8 ± 14.8	80.2 ± 14.9	82.6 ± 13.0	83.0 ± 12.8	87.0 ± 16.1	88.8 ± 15.7
BMI (kg/m^2^)	28.4 ± 4.7	28.6 ± 5.1	30.0 ± 4.5	29.9 ± 4.5	31.2 ± 4.5	31.7 ± 4.9
LM (kg)*	50.0 ± 11.3	50.3 ± 11.2	47.7 ± 9.1	48.5 ± 9.6	49.7 ± 12.3	54.0 ± 13.5^§^,†
SMM (kg)*	29.5 ± 7.2	30.2 ± 7.8	27.9 ± 5.8	28.3 ± 6.2	29.7 ± 7.1	31.3 ± 7.3†
FM (kg)¶	26.8 ± 10.2	26.9 ± 10.5	32.0 ± 10.5	29.1 ± 11.9	33.3 ± 9.8	34.1 ± 10.6
BF (%)*	33.3 ± 9.9	33.2 ± 10.4	38.2 ± 9.9	36.1 ± 10.5†	38.2 ± 8.3	37.5 ± 9.7
FFM (kg)*	53.1 ± 12.0	53.3 ± 11.9	50.6 ± 9.7	51.5 ± 10.3	53.7 ± 11.8	56.0 ± 12.1^#^

Data are presented as mean and standard deviation ( ±); BF = body fat; BMI = body mass index; FFM = fat-free mass; FM = fat mass; LM = lean mass; SMM = skeletal muscle mass; * = time effect between first and second assessment (*p* < 0.05); ¶ = group effect with lower values for mild group *vs.* severe/critical group (*p* < 0.05); § = interaction effect between time and group (*p* < 0.05); # = interaction effect between time and group (*p* < 0.05); † = a intra-group difference (*p* < 0.05).

A time effect was observed for all groups, with a significant increase in LM (*p* = 0.003; ƞ^2^_p_ = 0.10—*moderate*), SMM (*p* = 0.002; ƞ^2^_p_ = 0.12—*moderate*), and FFM (*p* < 0.001; ƞ^2^_p_ = 0.19—*large*); and a significant reduction in BF (*p* = 0.014; ƞ^2^_p_ = 0.07—*small*).

A time-group interaction was observed for LM (*p* = 0.025; ƞ^2^_p_ = 0.09—*moderate*), with higher values for the severe/critical group after 1 year (*p* = 0.003); and FFM (*p* = 0.046; ƞ^2^_p_ = 0.07—*moderate*), with higher values in the severe/critical group after 1 year (*p* = 0.001). After time effect analysis, a t-test was performed to identify possible intra-group differences, with the severe/critical group showing a significant increase in LM (*p* = 0.017; *d* = 0.47, 95% CI 0.08 to 0.85—*small*), SMM (*p* < 0.001; *d* = 0.92, 95% CI 0.47 to 1.37—*large*) and FFM (*p* < 0.001; *d* = 0.79, 95% CI 0.35 to 1.22—*moderate*), but no significant differences were observed in BF (*p* > 0.05). The moderate group significantly decreased BF (*p* < 0.003; *d* = 0.68, 95% CI − 1.08 to − 0.22—*moderate*). No significant differences were found in LM, SMM, BF, and FFM for mild and moderate groups (*p* > 0.05).

There was a group effect for FM (*p* = 0.043; ƞ^2^_p_ = 0.08—*moderate*), with higher values in the severe/critical group compared to the mild group (*p* = 0.037). There were no group, time, or interaction effects for body mass and BMI (*p* > 0.05).

### Bruce test

Table 4 shows the parameters from the Bruce test.

**Table 4:** Parameters analyzed in the Bruce test of study participants.

Variables	Mild (*n* = 29)		Moderate (*n* = 32)		Severe/critical (*n* = 29)	
	Before	After	Before	After	Before	After
VO_2_ peak (mL/kg/min)	27.1 ± 9.4	26.2 ± 8.0	21.7 ± 8.9	23.2 ± 7.5	23.0 ± 5.5	23.8 ± 6.8
Distance walked (m)*	728.6 ± 291.1	743.8 ± 371.7	566.3 ± 254.8	618.4 ± 271.1	546.6 ± 222.3	619.7 ± 247.4
HR peak (bpm)	166 ± 23	162 ± 19	154 ± 25	154 ± 26	157 ± 24	159 ± 21
Final SpO_2_ (%)†	96.0 ± 3.1	94.2 ± 3.5	95.2 ± 2.6	95.5 ± 2.0	92.2 ± 3.8	92.5 ± 4.7
Final SBP (mmHg)	158.6 ± 18.1	159.4 ± 19.0	151.6 ± 25.3	153.6 ± 21.6	159.3 ± 14.8	159.0 ± 23.1
Final DBP (mmHg)#	78.5 ± 7.6	82.9 ± 13.3	90.0 ± 13.2	86.6 ± 11.5	89.5 ± 14.3	86.6 ± 12.8
RQ peak	1.1 ± 0.2	1.1 ± 0.2	1.1 ± 0.1	1.2 ± 0.2	1.1 ± 0.1	1.3 ± 0.3§
RPE peak	16 ± 3	17 ± 2	17 ± 3	16 ± 3	16 ± 3	17 ± 2

Data are presented as mean and standard deviation ( ±); DBP = diastolic blood pressure; HR = heart rate; SBP = systolic blood pressure; RPE = rating of perceived exertion; RQ = respiratory coefficient; SpO_2_ = peripheral oxygen saturation; VO_2_ peak = peak oxygen consumption; * = time effect between first and second assessment (*p* < 0.05); † = group effect with lower values for severe/critical group *vs.* mild and moderate groups (p < 0.05); # = group effect with lower values for mild group *vs.* moderate and severe/critical groups (*p* < 0.05); § = interaction effect between time and group (*p* < 0.001); ¶ = a intra-group difference (*p* < 0.05).

A time effect was observed for all patients about the distance walked (*p* = 0.037; ƞ^2^_p_ = 0.05—*small*), with an increase after 1 year. However, the t-test showed a significant difference in the severe/critical group (*p* = 0.017; *d* = 0.47, 95% CI 0.08 to 0.85—*small*). Mild and moderate groups did not present a significant difference for distance walked (*p* > 0.05). A group effect was observed for SpO_2_ (*p* = 0.043; ƞ^2^_p_ = 0.08—*moderate*), with lower values in the severe/critical group compared to mild (*p* = 0.002) and moderate (*p* < 0.001) groups. A group effect was observed for DBP (*p* = 0.005; ƞ^2^_p_ = 0.12—*moderate*), with lower values in the mild group compared to the moderate (*p* = 0.008) and severe/critical (*p* = 0.018) groups. A time-group interaction was observed for RQ (*p* < 0.001; ƞ^2^_p_ = 0.31—*large*), with significantly higher values in the severe/critical group (*p* = 0.003) after 1 year. However, no time, group, or interaction effects were observed for peak VO_2_ peak, HR peak, final SBP, or RPE (*p* > 0.05).

### Monitoring vital responses after the Bruce test

**Figure 2:**
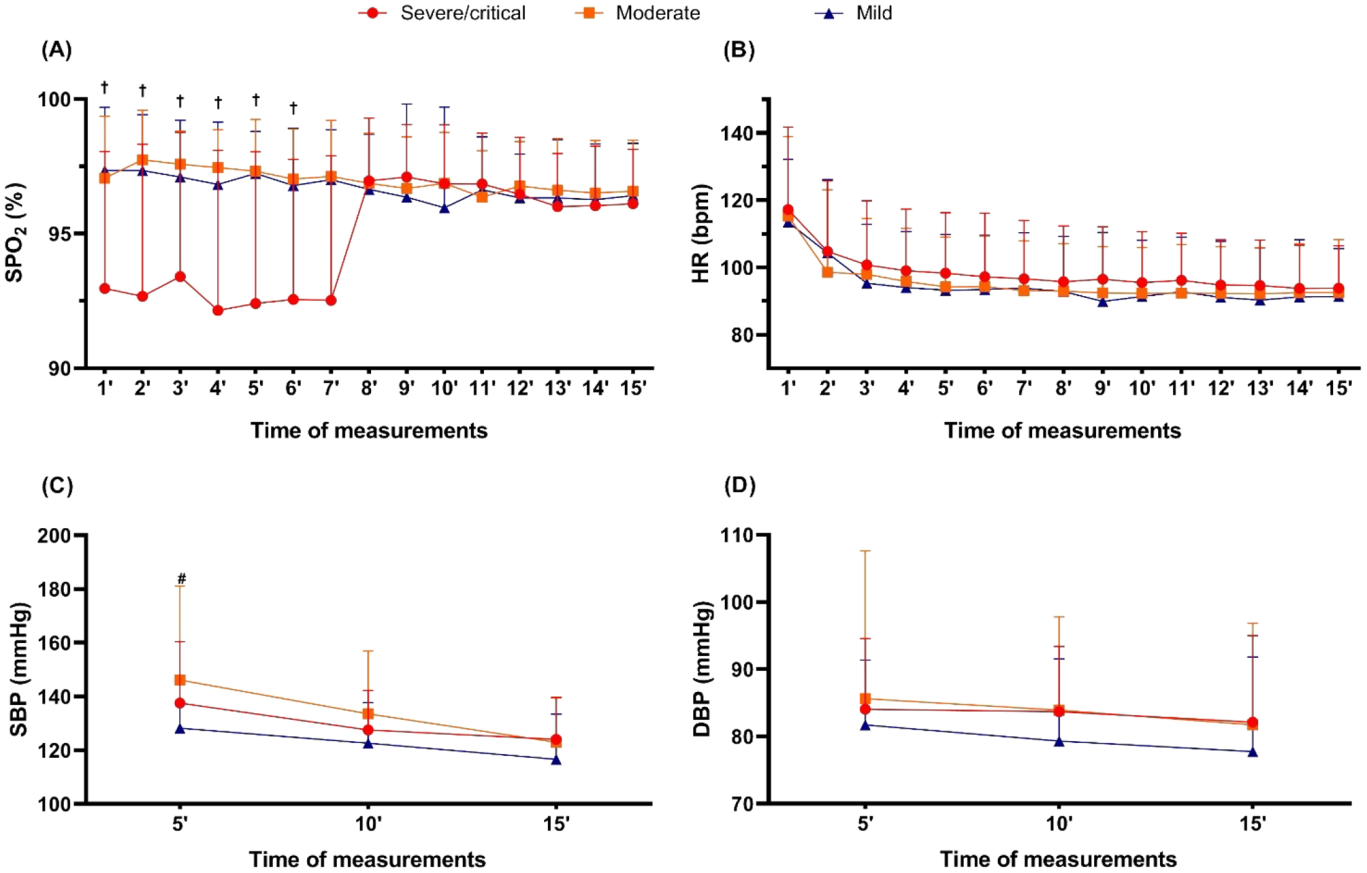
Peripheral oxygen saturation, heart rate, systolic and diastolic blood pressure after Bruce testing at different times in the post-COVID-19 survivor groups. Panel (**A**) = SPO_2_ (peripheral oxygen saturation for 15 min after the test); Panel (**B**) = HR (heart rate for 15 min after the test); Panel (**C**) = SBP (systolic blood pressure, 5 min after the test, 10 min after the test and 15 min after the test); Panel (**D**) = DBP (diastolic blood pressure, 5 min after the test, 10 min after the test and 15 min after the test); † = group difference (*p* < 0.05, severe/critical group *vs* moderate and mild groups); # = Time difference with higher values 5 min after test when compared to 15 min after test (*p* < 0.05).

Figure 2 shows the SpO_2_, HR, SBP, and DBP monitoring from 1 to 15 min after the stress test. For the post-stress test, SpO_2_, we observed a time-group interaction (*p* < 0.001; ƞ^2^_p_ = 0.16—*large*) from 1 to 6 min after the test, with lower values in the severe/critical group compared to mild (*p* < 0.001) and moderate (*p* < 0.001) groups.

Also, a group effect for SpO_2_ was detected (*p* < 0.001; ƞ^2^_p_ = 0.20—*large*), with lower values in the severe/critical group compared to mild (*p* < 0.001) and moderate (*p* < 0.001) groups.

For the post-stress test SBP, a group effect was observed (*p* = 0.024; ƞ^2^_p_ = 0.08—moderate), with lower values in the mild group than in the moderate group (*p* = 0.02). Post-stress test HR and DBP had no group, time, or interaction effects (*p* > 0.05).

## Discussion

The present study aimed to analyze the self-reported sequelae after COVID-19 and possible changes in body composition and cardiorespiratory fitness after 1 year. The main outcomes observed were (i) the most recurrent persistent symptoms were related to memory deficit, fatigue, difficulty concentrating, dyspnea, and capillary loss, although there were no differences between the severity of disease; and (ii) increasing LM, SMM, and FFM in the severe/critical group after 1-year; (iii) a group effect with lower values of BF in the FM mild group when compared to the severe/critical group; (iv) increase in the distance walked in the Bruce test just for the severe/critical group after 1 year; (vi) a group effect for DBP post-Bruce test with lower values for mild group when compared to moderate and severe/critical groups. No significant differences were observed for the anthropometric and other ergospirometric and hemodynamic variables.

### Long COVID symptoms

The persistent symptoms of COVID-19 require early intervention to minimize possible sequelae in different severity symptoms, i.e., mild, moderate, and severe/critical patients with follow-up. Asymptomatic patients also require follow-up, especially those with some vulnerable condition or associated comorbidities, combined with multidisciplinary actions to promote better outcomes given the reported sequelae^22,23^.

### Body composition responses in long COVID patients

Patients with greater loss of LM 6 months after contracting COVID-19 could not recover muscle health^24^, and unplanned hospitalizations tend to promote a reduction in upper limb muscle strength, LM, limb muscle strength, maximum isometric handgrip strength, and of one-repetition maximum in the extensor chair^25,26^. Long COVID-19 patients also have a decreased LM compared to a control group (without a diagnosis of COVID-19)^23^. However, the present study showed increased LM, SMM, and FFM for severe/critical patients after 1 year. Discrepancies in the measurement time between the present study and the others may justify this finding. In this regard, there was a need for medical clearance of the present study participants to perform submaximal exercise tests, and the recovery time will depend on each patient and possible limitations sequels.

The convalescent period after infection is susceptible to different complications, especially with distinct immunological signatures that open an "immunological window," allowing the development of complications such as acute myocardial infarction and myocarditis, among other clinical manifestations^27,28^. Therefore, the morphophysiological parameters were collected after recovery from the acute sequelae recorded within the subdivision of symptoms (mild, moderate, and severe/critical). Another study indicates that long COVID-19 patients showed significant improvement in muscle strength, mobility, and cardiorespiratory fitness after 12 months of in-person physical therapy rehabilitation^29^.

Previous studies found that BF was higher in long COVID patients compared to a control group (i.e., without a diagnosis of COVID-19), and outpatients had a lower FM compared to patients hospitalized for COVID-19 with the same BMI^9,26^. Considering the difference in FM between groups persisted after 1 year (mild with lower FM *vs* severe/critical cases), actions to provide improvement in body composition, with reduction of FM, BF, and increase in LM, remain substantial for improving the body composition of COVID-19 survivors. Concurrent training may be a relevant strategy for improving health-related physical fitness by increasing muscle strength, cardiorespiratory fitness, and LM, in addition to reducing FM^30^. Rehabilitation sessions via aerobic and resistance exercise should control volume, intensity, density, frequency, and progression based on each clinical case, as well as on the physical fitness indicators that were most affected by the disease^20,30^.

### Cardiorespiratory responses in long COVID patients

VO_2_ peak, HR peak, and RPE did not differ between groups, and there was no difference between the times (at baseline *vs* after 1 year), suggesting that the intensity was similar. Significant differences between outpatients and severe/critical patients were found previously^9^. The absence of differences between the groups in the present study may be related to a reduction in endothelial damage during convalescence and a subsequent return to activities of daily living for severe/critical patients^9,31^. However, the self-reported level of physical activity of patients with different symptoms did not differ.

The distance walked in the Bruce test increased the severe/critical group after 1 year, indicating an improvement in physical fitness and a possible reduction in sequels provoked by COVID-19 survivors. Similar responses were identified in another study, with a significant increase in the distance walked in the 6-min walk test 12 months after hospital discharge^30^. In the present study, less than 50% of the patients (mild, moderate, and severe/critical) reported being physically active, i.e., > 150 min of physical activity/week. However, the improvement in patients' cardiorespiratory fitness is also associated with the physical reconditioning of individuals who returned to their respective activities of daily living, in addition to a possible reduction in residual inflammation and organic damage (this condition was not analyzed in the present study)^9,31^.

Considering the increased distance walked during the Bruce test, an interaction was also observed with higher values for the RQ of severe/critical patients after 1 year, suggesting an improvement in high exercise tolerance in long duration, justified by the increased intolerance of exercise intensity^29^. Final SpO_2_ post-Bruce test showed a group effect, with significantly lower values for severe/critical patients that may be related to chronic hypoxemia after physical effort or even to vascular and pulmonary changes and a decrease in pulmonary function^10,31,32^.

The main signals that affect respiratory control are derived from the response of peripheral chemoreceptors and mechanoreceptors, in addition to an abnormal muscular effort of thoracic muscles and a reduction in lung compliance, accentuating dyspnea that affects performance in the effort^33^. Cardiorespiratory rehabilitation of these patients requires monitoring of SpO_2_, blood pressure, and cardiac function in addition to the application of the principles of interdependence between volume and intensity, increasing loads, biological individuality, and periodic assessments for evaluation of outcomes and mitigation of possible sequelae of COVID-19^34^.

### Blood pressure responses in long COVID patients

Post-Bruce test DBP test also differed among long COVID-19 patients, with higher values for the moderate and severe/critical group than for the mild group. DBP response during physical exertion is related to comorbidities (prevalence of obesity, systemic arterial hypertension, diabetes mellitus, and tobacco use) but is not independently associated with a higher risk of death from cardiovascular diseases^35^. A systematic review with meta-analysis identified that SBP ≥ 210 mmHg for males and ≥ 190 mmHg for females in moderate effort intensity can be considered an independent risk factor for cardiovascular events and increased mortality^36^. The physiology and pathophysiology of DBP after physical effort have not yet been fully elucidated, and the increase in DBP may be concatenated to greater peripheral arteriolar resistance, increased afterload, and even arterial stiffness or dysfunction, and early signs of atherosclerotic vascular disease^35^.

A high DBP response to physical effort is a predictive factor for increased systemic arterial hypertension. Because systemic arterial hypertension can often be asymptomatic, patients can progress to structural and/or functional changes in target organs and endothelial dysfunction, with an imbalance between vasodilating and vasoconstrictor substances affecting vascular function, with reduced blood pressure compliance capacity of the great arteries, impairing pressure homeostasis^37,38^. A study observed a significant increase in SBP and DBP in males and females during the pandemic period (between 2019 and 2020)^39^, probably associated with increased alcohol consumption, weight gain, lower level of physical activity, emotional stress, and less continuous medical care (with reduced medication adherence), although the parameters above were not observed and/or measured in the present study, since the collections were performed strictly during the pandemic period. However, the increase in SBP during physical exercise is a normal response related to the intensity of effort^40^.

### Limitations, strengths, and future directions

This study has some limitations. First, the lack of follow-up during the acute infection of the patients is justified by the intolerance to exercise. Second, there was no follow-up after the 1 year between the evaluations, and behavior changes (e.g., physical activity and nutrition habits) and other features not accessed in the study might be associated with improving body composition and cardiopulmonary fitness in the hospitalized groups. Third, the loss of follow-up in the second evaluation may have impacted the results; however, loss rates were similar in all groups. Unfortunately, these patients opted not to return for re-assessment, and this was related to (i) lack of time; (ii) not understanding the necessity to perform a re-assessment; (iii) patients believe they are completely recovered from COVID; and (iv) lack of financial resources to travel to university and not work part-time. Considering the future perspectives for research, Patients may be evaluated over months and even years to understand pathophysiological responses. Furthermore, actions that seek to assess, intervene, and re-evaluate long COVID patients associated with a control group (without the disease) may guide more assertive rehabilitation actions.

### Clinical applications

Given clinical relevance, some points can be highlighted: (i) hospitalized patients need to be monitored periodically on body composition, cardiorespiratory fitness, and vital signs; (ii) all COVID-19 survivors independently of the disease severity can be monitored about fatigue, dyspnea, muscle pain, joint pain, dizziness, tinnitus, sensation of hearing loss, otalgia, ageusia, anosmia, memory deficit, difficulty concentrating and capillary loss and (iii) earlier interventions with health professionals can reduce the possible impacts (sequels) of COVID-19.

## Conclusions

Cardiopulmonary fitness parameters did not differ among the groups, but severe/critical cases maintained worse hemodynamic responses to exercise. Fat mass showed higher values in severe/critical cases than in mild cases in which excess adiposity is related to low-grade inflammation. Considering these responses, regular physical activity and healthy nutrition programs are fundamental for all long COVID patients (biological individuality, symptoms, possible limitations, among other aspects). The prevalence of long-term symptoms among the groups was not different either. Neurocognitive dysfunction, fatigue, and dyspnea are the most prevalent long-term symptoms. Regardless of the severity of COVID-19, it is essential to reassess and identify the most prevalent long-term sequelae so that more precise health promotion interventions can be performed.

## Data Availability

The datasets generated during and/or analyzed during the current research are available from the corresponding author upon reasonable request.

## Abbreviations

ANOVA: Analysis of variance
BF: Body fat
BM: Body mass
BMI: Body mass index
DBP: Diastolic blood pressure
FFM: Fat-free mass
FM: Fat mass
HR: Heart rate
ICU: Intensive care unit
LM: Lean mass
RPE: Rating of perceived exertion
RQ: Respiratory coefficient
SBP: Systolic blood pressure
SMM: Skeletal muscle mass
SpO_2_: Peripheral oxygen saturation
VCO_2_: Carbon dioxide production
VE: Minute-ventilation
VO_2_: Oxygen consumption
